# Evaluating Standards in Psychiatric Admission Processes

**DOI:** 10.1192/bjo.2025.10545

**Published:** 2025-06-20

**Authors:** Basil Aldweik, Monsur Badmus, Balaji Wuntakal, Neil Kotecha

**Affiliations:** Royal College of Psychiatrists, Portsmouth, United Kingdom

## Abstract

**Aims:** For the first QIP our objective was by August 2024, a completion of admission checklists jobs on clerking by out of hours Doctors in Solent and Southernhealth Foundation Trusts Psychiatric Inpatient Units by 100%.

However, if tasks were not completed, we would be expecting documentation of the reason why on SystmOne tabbed Journal and Rio progress notes (electronic records).

**Methods:** For the first QIP, 30 patients were randomly selected from current admissions in three facilities: The Limes (10 patients), The Orchards (Maples and Hawthorn, 5 patients each) and Elmliegh Hospital (10 patients).

For the completed audit Cycle, 35 patients were randomly selected from current admissions in four facilities: The Limes (10 patients), The Orchards (Maples and Hawthorn, 5 patients each), Elmleigh Hospital (10 patients), and Ravenswood House Forensic Hospital (5 patients).

The audit involved reviewing clerking documentation on SystmOne and Rio, measuring performance indicators for the completion of admission tasks, including DNACPR, mental capacity assessments, mental state examinations, physical examination, current medications, allergies and sensitivities, and VTE assessments.

During our first QIP, a guide was introduced in the trainee handbook to demonstrate how to complete an admission on S1 in addition to a video demonstration, link shared with all doctors working within the trust.

**Results:** There was excellent compliance with admission tasks across most wards, with a 100% completion rate in the Limes, The Orchards and Ravenswood House. Key achievements included successful documentation of physical examinations, current medications, allergies, and sensitivities. The audit demonstrated substantial progress in standardising admission documentation processes.

**Conclusion:** The audit results showed excellent overall compliance in completing admission tasks across most wards, with particular success in completing physical examinations, current medications, allergies, and sensitivities documentation. This audit reflects significant progress in standardising admission documentation across psychiatric inpatient units. This audit’s findings will support continued improvements in admission processes, enhancing both compliance and patient safety. The results have been disseminated trust wide and incorporated as part of the junior doctor induction programme.

